# A multicentre clinical study on the injection of ceftriaxone/sulbactam compared with cefoperazone/sulbactam in the treatment of respiratory and urinary tract infections

**DOI:** 10.1186/1476-0711-12-38

**Published:** 2013-12-09

**Authors:** Xiaojuan Xin, Li Jian, Xiaoying Xia, Bei Jia, Wenxiang Huang, Chongzhi Li, Changzheng Wang, Lixin Zhou, Xiuzhen Sun, Xinghuo Tang, Yijiang Huang, Yunkui Zhu, Weili Zhang

**Affiliations:** 1 Key Laboratory of Infectious and Parasitic Diseases in Chongqing, Department of Infectious Diseases, the First Affiliated Hospital of Chongqing Medical University, No. 1 Youyi Road, Yu Zhong District, Chongqing 400016, China; 2 The people’s Hospital of Dazu District, Chongqing, China; 3 Secondary Affiliated Hospital, Third Military Medical University, Chongqing, China; 4 Renji Hospital, Shanghai Jiao Tong University School of Medicine, Shanghai, China; 5 Second Affiliated Hospital, Medical School of Xi’an Jiaotong University, Xi’an, China; 6 First Affiliated Hospital, Guangxi Medical University, Nanning, China; 7 Hainan General Hospital, Haikou, China; 8 Lanzhou General Hospital of Lanzhou Military Area Command of Chinese PLA, Lanzhou, China; 9 Second Affiliated Hospital, Chongqing Medical University, Chongqing, China

**Keywords:** Resistant bacterial infection, Ceftriaxone/sulbactam, Multicentre clinical study

## Abstract

**Objective:**

This clinical study was designed to evaluate the efficacy and safety of this therapy in the treatment of respiratory and urinary infections caused by ceftriaxone-resistant bacteria in comparison with the effect of cefoperazone/sulbactam on cefoperazone-resistant bacteria.

**Methods:**

A total of 285 patients aged from 18 to 65 years old, with a respiratory or urinary tract bacterial infection, were enrolled into this multicentre, open-label, controlled clinical study, and bacteria that were either ceftriaxone-resistant or cefoperazone-resistant were isolated from the patients, whose condition had not improved after three days of treatment with ceftriaxone or cefoperazone. To be selected for the study, bacterial cultures obtained from the patients had to be positive before enrolment, and all of the isolates were required to be β-lactamase-positive. Of these patients, 253 completed the trial, and 263 were enrolled into the intention-to-treat (ITT) analysis. All of the 285 patients were included in the safety analysis.

**Results:**

The cure and effective rates were 39.55% and 85.07% in the ceftriaxone/sulbactam group and 36.43% and 79.84% in the cefoperazone/sulbactam group; the bacterial eradication rates were 83.58% and 83.72%; and the adverse-event rates were 7.48% and 7.80%, respectively. There were no significant differences between the two groups (p > 0.05).

**Conclusion:**

Ceftriaxone/sulbactam is as effective and well-tolerated as cefoperazone/sulbactam for the treatment of intermediate and severe bacterial infections caused by resistant strains.

## Introduction

Ceftriaxone was used in clinical practice for approximately 30 years and was the first third-generation cephalosporin approved for the once-daily treatment of patients with gram-positive or gram-negative bacterial infections. However, based on data obtained from the Chinese Ministry of Health National Antibacterial Resistance Surveillance Net (Mohnarin), the majority of the isolates detected (except *Streptocococcus* spp., *Proteus mirabilis* and *Salmonella* spp.) were highly resistant to ceftriaxone with a resistance frequency ranging from 35%-70% [1].

Sulbactam is a molecule that is administered in combination with β-lactam antibiotics to overcome the effects of β-lactamase. It is an irreversible inhibitor of β-lactamase that binds to the enzyme and prevents it from interacting with the antibiotic. Sulbactam is able to inhibit the most common forms of β-lactamase and has been commercially available in combination with either ampicillin or cefoperazone [2].

The addition of sulbactam to ceftriaxone treatment not only augments the activity of ceftriaxone against β-lactamase-producing bacteria but also maintains its cost-effectiveness (once or twice daily administration). A number of studies have been conducted on the pharmacokinetics or *in vitro* susceptibility of this combination [3-7]. However, few clinical trials have reported on the effects of using this new agent for the treatment of respiratory and urinary infections caused by ceftriaxone-resistant bacteria.

Our study was undertaken to evaluate the clinical efficacy and safety of injection with a fixed ratio (4:1) of this β-lactam/β-lactamase inhibitor combination, as well as a comparison to cefoperazone/sulbactam (2:1) injection, in the treatment of respiratory and urinary tract infections caused by resistant bacteria. This investigation was conducted at eight medical centres in China over 5 years.

## Patients and methods

### Study design

This multicentre, open-label, single-blinded, controlled clinical trial was undertaken in eight Chinese teaching hospitals from 2005 to 2009. All of the cases were of intermediate or severe infection caused by ceftriaxone-resistant or cefoperazone-resistant bacteria. The protocol was approved by the appropriate ethics committees in each hospital and conducted in compliance with the Declaration of Helsinki.

### Key inclusion criteria

The selected inpatients or outpatients ranged in age from 18 to 65 years old. They exhibited evidence of a respiratory or urinary tract bacterial infection that required antimicrobial treatment for more than three days. Their condition had not improved after three days of treatment with ceftriaxone or cefoperazone. To be selected for the study, bacterial cultures obtained from the patients had to be positive before enrolment, and all of the isolates were required to be β-lactamase-positive. In patients selected for the treatment group, the isolates were resistant to ceftriaxone but sensitive to ceftriaxone/sulbactam, whereas in patients selected for the control group, the isolates were resistant to cefoperazone but sensitive to cefoperazone/sulbactam. An informed consent statement, which was approved by the local ethical review board, was signed before enrolment in the trial. The diagnosis and judgment of the condition of bacterial infection were based on the symptoms, signs and related laboratory examinations. The patients included in the study who suffered from respiratory tract infections (RTIs) were required to exhibit radiographic evidence and at least two of the following symptoms: fever (>37°C) or hypothermia, cough with sputum, dyspnea, crackles, rales, pleuritic pain, dullness of percussion, and leucocytosis or leucopenia. Patients suffering from urinary tract infections (UTIs) were eligible for inclusion if they experienced at least two of the following symptoms: fever, urinary irritation symptoms (dysuria, frequency, and urgency), suprapubic pain, costovertebral tenderness or flank pain, or the presence of more than five leucocytes per high-power field in a centrifuged sediment. The judgement of severity of respiratory or urinary tract conditions was based on symptoms, signs and laboratory examinations of the patients according to the criteria from Practice of Internal Medicine [8].

### Key exclusion criteria

Patients were excluded from the study if they had a history of previous allergy to β-lactam antibiotics, had participated in a new drug trial in the previous three months, had a history of severe cardiac, renal or haematological impairment, or had increased hepatic enzyme levels >2 times the upper limit of the normal range. Patients with a terminal malignancy or psychiatric illness, as well as women who were pregnant or nursing, were also excluded.

### Drug administration

Patients were assigned to receive intravenous injections with either 2.5 g of ceftriaxone/sulbactam or 4.5 g of cefoperazone/sulbactam twice daily for 7–14 days.

### Assessment and monitoring

Clinical assessments, including the symptoms and signs, of all of the patients were performed throughout the medication period. Laboratory assessments, including routine haematology, chemistry and urinalysis profiles, electrocardiogram, chest X-rays and cultures of expectorated sputum or urine were performed at the time of enrolment and one day after the completion of the therapy. The safety and tolerability were assessed by observation and by volunteered reports from the patients. Adverse events were documented with the concomitant medications. Women of childbearing potential were required to exhibit normal menstruation and a negative pregnancy test. The patients with a cured or markedly improved clinical outcome were followed up 7 days after the treatment for microbiological, efficacy and safety assessments.

Specimens were isolated from the sputum or urine for bacterial culture prior to the initial treatment, on the first day of treatment and on the 7^th^ day after the completion of the therapy. The Kirby-Bauer disc diffusion method and the minimum inhibitory assay (broth dilution method) were conducted to test the susceptibility of all of the isolates to six antimicrobials, including ceftriaxone, ceftriaxone/sulbactam, cefoperazone, cefoperazone/sulbactam, cefepime and imipenem/cilastin. The judgment of microbial susceptibility was determined according to the CLSI 2005–2009 guidelines, and the susceptibility rate was calculated based on standards [9]. The minimum inhibitory concentrations of all of the isolates were measured at the laboratory of the First Affiliated Hospital of Chongqing Medical University.

The clinical efficacy was classified as follows:

• cure – all of the presenting symptoms and signs were resolved, and the laboratory tests and clinical procedures were normal;

• marked improvement – only one abnormality remained;

• improvement – at least two abnormalities remained at the termination of treatment;

• failure – clinical manifestations remained or were aggravated after 72 h of treatment.

The proportion of the ‘cure’ and ‘marked improvement’ categories was employed to calculate the overall efficacy rate.

The bacteriological response was evaluated as follows: the intervention was considered to be successful if it achieved complete eradication (elimination of the original causative pathogens); the intervention was considered unsatisfactory if only partial or no eradication, superinfection or re-infection were found. The bacteriological response was defined as assumed eradication if there were no materials for culture in the patients who had suffered from respiratory tract infection at the end of the treatment and/or the follow-up visit.

The safety assessments were classified into five categories: ‘definitely’, ‘probably’ or ‘possibly’ drug-related, and ‘possibly’ or ‘definitely’ drug-unrelated. The proportion of the three drug-related categories was used to calculate the side-effect rate.

### Data analysis

All of the statistical analyses were performed using the SAS software, version 6.12 (SAS Institute, Cary, N.C., USA). Student’s t test, χ^2^ or Fisher’s exact test were used to test the hypotheses, according to the type of the variants and the patient of study. The efficacy and safety analyses were based primarily on the full analysis set (FAS) population, which included all of the patients who had received at least one dose of the study medication and had the study disease.

## Results

### Patient disposition and baseline characteristics

**Figure 1:**
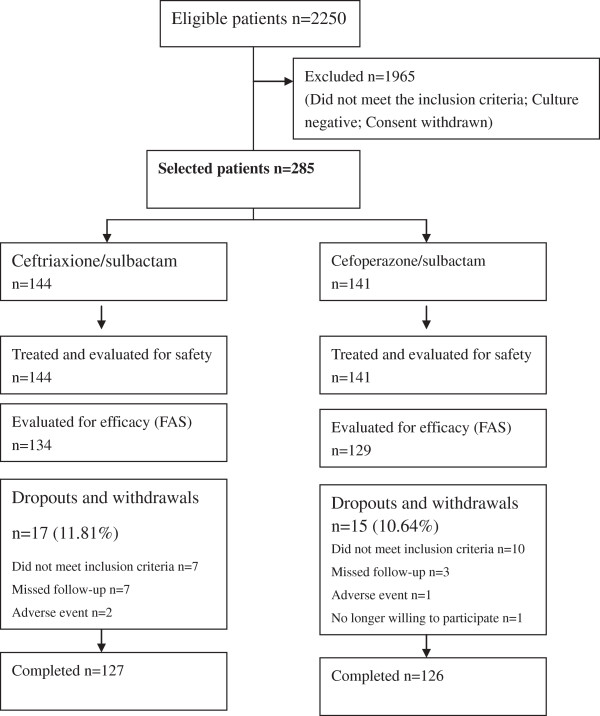
Subject disposition.

After screening 2250 patients for entry into the study, 144 patients were selected to receive ceftriaxone/sulbactam, and 141 received cefoperazone/sulbactam (Figure 1). Between the two groups, there were no differences in age, gender, weight, course of disease, condition of infection, temperature before enrolment, presence of underlying diseases or type of infection (Table 1).

**Table 1:** The demographic and baseline characteristics

**Characteristics**	**Ceftriaxone-sulbactam**	**Cefoperazone-sulbactam**	***P***
Male, n(%)	64(44.44%)	63(44.68%)	0.9680
Female, n(%)	80(55.56%)	78(55.32%)	
Mean age, years(SD)	46.00±15.86	46.47±14.49	0.9370
Mean weight, kg(SD)	59.36±11.08	59.61±11.29	0.9742
Treatment duration	8.45±3.03	8.10±3.00	0.3251
Condition			
Intermediate	113	116	0.7901
Severe	31	25	0.5085
Temperature(°C)	37.19±0.79	37.04±0.77	0.0840
With underlying diseases	34	29	0.6209
Type of infection, n(%)			
Respiratory tract infection			0.2348
Pneumonia	33(45.83%)	32(44.44%)	
Exacerbation of chronic bronchitis	21(29.17%)	15(20.83%)	
Bronchiectasis	12(16.67%)	11(15.28%)	
Others	6(8.33%)	14(19.44%)	
Urinary tract infection			0.8021
Acute pyelonephritis	24(33.33%)	19(27.54%)	
Exacerbation of chronic pyelonephritis	9(12.50%)	7(10.14%)	
Acute cystitis	33(45.83%)	37(53.62%)	
Others	6(8.33%)	6(8.70%)	

SD: Standard deviation.

### Clinical efficacy

Although the overall clinical efficacy was higher in the ceftriaxone/sulbactam group than in the cefoperazone/sulbactam group, the difference was not statistically significant. There was a total cure rate of 39.55% (53/134) in the ceftriaxone/sulbactam group, compared with 36.43% (47/129) of those receiving cefoperazone/sulbactam. The efficacy rate of each group was 85.07 and 79.84%, respectively. The cure and efficacy rates for respiratory and urinary tract infections in the ceftriaxone/sulbactam group were also not significantly different. Both of the antimicrobials were equally efficient against the infections caused by Gram-negative bacteria (*p* > 0.05). However, the efficacy rate for the diseases caused by gram-positive bacteria in the cefoperazone/sulbactam group was significantly higher than that in the ceftriaxone/sulbactam group (*p* < 0.05), although the sample size was less than 10 cases (Table 2).

**Table 2:** Summary of the clinical response

	**Ceftriaxone-sulbactam**	**Cefoperazone-sulbactam**	***P***
Clinical response in total at the end of therapy, n(%)	134	129	
Cure	53(39.55)	47(36.43)	0.6138
Cure + marked improvement	114(85.07)	103(79.84)	0.3300
Improvement + failure	20(14.93)	26(20.16)	
Clinical response for RTIs at the end of therapy, n(%)	65	65	
Cure	18(27.69)	13(20.00)	0.4107
Cure + marked improvement	50(76.92)	46(70.77)	0.5398
Improvement + failure	15(23.08)	19(29.23)	
Clinical response for UTIs at the end of therapy, n(%)	69	64	
Cure	35(50.72)	34(53.13)	0.8626
Cure + marked improvement	64(92.75)	57(89.06)	0.5513
Improvement + failure	5(7.25)	7(10.94)	
Clinical response caused by gram-negative germs at the end of therapy, n(%)	129	120	
Cure	53(41.09)	44(36.67)	0.5166
Cure + marked improvement	112(86.82)	94(78.33)	0.0935
Improvement + failure	17(13.18)13.18	26(21.67)	
Clinical response caused by gram-positive germs at the end of therapy, n(%)	5	9	
Cure	0(0.00)	3(33.33)	0.2582
Cure + marked improvement	2(40)	9(100)	0.0275
Improvement + failure	3(60)	0(0)	
Clinical response in total at the day 7 follow-up visit	114	103	
Continued resolution n(%)	114(100)	103(100)	
Relapse n(%)	0(0)	0(0)	

RTIs: Respiratory tract infections.

UTIs: Urinary tract infections.

### Microbiological efficacy

In total, 263 strains were evaluated, with 134 in the ceftriaxone/sulbactam group and 129 in the cefoperazone/sulbactam group. The overall eradication rate was 83.58% in the ceftriaxone/sulbactam group and 83.72% in the cefoperazone/sulbactam group. The bacteriological success rates for the overall and for the gram-negative or gram-positive pathogens were comparable between the treatment groups at the 7-day follow-up visit (Table 3).

**Table 3:** Summary of the microbiological response

	**Ceftriaxone-sulbactam**	**Cefoperazone-sulbactam**	***P***
Microbiological response on the day after completion of therapy, n(%)	1344	129	0.9347
Eradication	112(83.58)	108(83.72)	
Partial eradication/no eradication/super infection/re-infection	22(16.42)	21(16.28)	
Microbiological response for gram-negative germs, n(%)	129	120	0.8361
Eradication	109(85.83)	100(84.13)	
Partial eradication/no eradication/super infection/re-infection	18(14.17)	20(16.67)	
Microbiological response for gram-positive germs, n(%)	5	9	0.2269
Eradication	3(60.00)	8(88.89)	
Partial eradication/no eradication/super infection/re-infection	2(40.00)	1(11.11)	
Microbiological response for RTIs, n(%)	65	65	1.0000
Eradication	52(80.00)	53(81.54)	
Partial eradication/no eradication/super infection/re-infection	13(20.00)	12(18.46)	
Microbiological response for UTIs, n(%)	69	64	1.0000
Eradication	60(86.96)	55(85.94)	
Partial eradication/no eradication/super infection/re-infection	9(13.04)	9(14.06)	
Microbiological response at the day 7 follow-up, n(%)	114	103	0.8300
Eradication	80(70.18)	76(73.79)	
Partial eradication/no eradication/super infection/re-infection	4(3.51)	3(2.91)	
Assumed eradication(no material for culture)	30(26.32)	24(23.30)	

RTIs: Respiratory tract infections.

UTIs: Urinary tract infections.

In the ceftriaxone/sulbactam group, there were 129 ceftriaxone-resistant Gram-negative strains isolated. The eradication rate was 84.50% (109/129). Five ceftriaxone-resistant gram-positive strains were isolated, including 3 *Staphylococcus haemolyticus* (methicillin-sensitive) and 2 *Staphylococcus aureus* (methicillin-sensitive). The eradication rate was 60% (3/5).

In the cefoperazone/sulbactam group, there were 120 cefoperazone-resistant Gram-negative strains isolated. The eradication rate was 83.33% (100/120). Nine cefoperazone-resistant gram-positive strains were isolated, including 6 *Staphylococcus haemolyticus* (methicillin-sensitive) and 3 *Staphylococcus aureus* (methicillin-sensitive). The eradication rate was 88.89% (8/9).

For the respiratory tract diseases, there were 65 strains isolated from the ceftriaxone/sulbactam group, with a total eradication rate of 80.00% (52/65). The eradication rate was 75% (3/4) for the Gram-positive strains and 80.32% (49/61) for the gram-negative strains. There were 65 isolates from the cefoperazone/sulbactam group, with a total eradication rate of 81.53% (53/65). The eradication rate was 85.71% (6/7) for the gram-positive strains and 81.03% (47/58) for the gram-negative strains.

For the urinary tract diseases, there were 69 and 64 strains isolated from the ceftriaxone/sulbactam and cefoperazone/sulbactam groups, with total eradication rates of 86.96% (60/69) and 85.94% (55/64), respectively. The eradication rate for the gram-negative organisms was 88.24% (60/68) in the ceftriaxone/sulbactam group and 85.48% (53/62) in the cefoperazone/sulbactam group. Only one gram-positive strain was isolated from the ceftriaxone/sulbactam group, and it was not cleared. Two gram-positive strains were isolated and cleared in the cefoperazone/sulbactam group.

No significant difference was detected between the groups in the above eradication rates (*p* > 0.05).

The post-therapy (day 7 follow-up visit) bacteriological evaluation of the patients from the cured or marked improvement groups revealed no differences compared with the results at the end of therapy.

### In-vitro drug susceptibility assay

The K-B disc diffusion test results showed that the sensitivity rates of the isolates given ceftriaxone/sulbactam and cefoperazone/sulbactam were 84.88 and 86.73%, respectively. The difference was not statistically significant.

Minimum inhibitory concentration assays were conducted on all of the isolates. The results, which are expressed as the range of minimum inhibitory concentrations and the concentrations required to inhibit 50% and 90% of the isolates (the latter only for isolates with more than 10 strains), are shown in Table 4. These findings suggested that ceftriaxone/sulbactam was as active as or slightly superior to cefoperazone/sulbactam against the Enterobacteriaceae; however, for *P. aeruginosa* and *A. baumannii*, ceftriaxone/sulbactam was twofold less active than cefoperazone/sulbactam.

**Table 4:** ***In vitro *** **susceptibility of the clinical isolates to 6 antimicrobials**

**Isolates**		**Cefoperazone-sulbactam**	**Cefoperazone**	**Ceftriaxone-sulbactam**	**Ceftriaxone**	**Imipenem**	**Cefepime**
	MICr	32–4	128–64	32–81	28–64	8–1	16–2
E. coli(153)	MIC_50_	16	64	8	64	1	4
	MIC_90_	32	128	32	128	4	16
	MICr	32–2	128–32	16–2	128–32	4–1	16–2
K. pneumoniae(22)	MIC_50_	8	64	4	64	1	4
	MIC_90_	32	128	16	128	2	8
	MICr	32–4	128–32	32–8	128–64	8–2	16–2
E cloacae(18)	MIC_50_	16	32	8	64	2	4
	MIC_90_	32	128	32	128	4	16
	MICr	32–8	128–64	16–4	128–64	4–1	8–1
E. aerogenes(13)	MIC_50_	8	64	16	64	1	2
	MIC_90_	32	128	32	128	2	8
Proteus spp(6)	MICr	8–4	64–32	4–2	32–16	2–1	8–1
	MICr	32–4	128–32	32–8	128–32	64–4	64–8
A. baumannii(12)	MIC_50_	8	32	8	32	8	16
	MIC_90_	16	128	32	128	64	64
	MICr	32–2	128–4	32–4	128–8	64–4	32–4
P. aeruginosa(10)	MIC_50_	8	32	16	64	4	8
	MIC_90_	32	128	32	128	32	32
S. aureus(5)	MICr	32–16	64–32	32–16	64–32	16–2	8–4
S. haemolyticus(9)	MICr	32–16	64–32	32–8	64	16–2	8–4

MIC: minimum inhibitory concentration; MIC50: Minimum Inhibitory Concentration required to inhibit the growth of 50% of organisms; MIC90: Minimum Inhibitory Concentration required to inhibit the growth of 90% of organisms; MICr: Range of MIC.

### Drug safety

Overall, both agents were well-tolerated by the patients in this study. Eighteen adverse events were observed in the patients receiving ceftriaxone/sulbactam (n = 18, 7.48%), and 11 were noted in the patients receiving cefoperazone/sulbactam (n = 11, 7.80%). Fifteen drug-related events were identified in the ceftriaxone/sulbactam group (15/144, 10.42%), and 10 were identified in the cefoperazone/sulbactam group (10/141, 7.09%). In the ceftriaxone/sulbactam group, the potentially drug-related clinical adverse reactions were as follows: rashes (1), headache (1), chest distress (1), nausea (2), and diarrhoea (1). The laboratory abnormalities included leucopenia (2) and hepatic dysfunction, which was characterised by a mild increase in the level of alanine transferase (7). In the cefoperazone/sulbactam group, 2 patients complained of rashes and 1 reported a headache. The laboratory abnormalities were leucopenia (3) and a slightly increased serum level of alanine transferase (4). The majority of the adverse events were mild to intermediate and spontaneously alleviated or recovered. There were 5 patients who discontinued the treatments due to adverse events, among whom two were in the ceftriaxone/sulbactam group (headache and severe renal haemorrhage) and three were in the cefoperazone/sulbactam group (headache, leucopenia and rashes). One serious adverse event was reported in the ceftriaxone/sulbactam group due to nephrorrhagia, which was confirmed to be unrelated to the study medication. The frequency of adverse events did not differ significantly between the two groups (p > 0.05).

## Discussion

China has one of the world’s highest reported rates of bacterial resistance. For cephalosporins, the most frequently used antimicrobials clinically, the emergence and prevalence of bacterial resistance, especially to the third-generation cephalosporins, has shifted the focus to the combination of β-lactam and β-lactamase inhibitors. The overuse of carbapenems and the subsequent increase in resistance to these drugs also warrants consideration of the role of the β-lactam and β-lactamase inhibitor combination. Cefoperazone/sulbactam is a conventional combination with activity against ESBL-producing pathogens and clinical efficacy in a variety of infections [2,10-12]. Therefore, cefoperazone/sulbactam was chosen as the control treatment for testing the efficacy and safety of ceftriaxone/sulbactam, a combination recently introduced in China. To verify the efficacy against resistant strains, all of the enrolled patients had respiratory or urinary infections with isolated bacteria that were resistant to ceftriaxone or cefoperazone.

Previous studies have suggested that a once-daily dose of ceftriaxone/sulbactam is sufficient to treat the infections caused by various organisms due to the high plasma-MIC levels of this combination [4]. However, when considering the features of bacteria and the dosage of cefoperazone/sulbactam, to guarantee full clinical efficacy, we chose to perform twice-daily injections of 2.5 g of ceftriaxone/sulbactam in this trial.

Our study on infections of the respiratory and urinary tracts further verified the clinical efficacy and safety of ceftriaxone/sulbactam. The results showed that ceftriaxone/sulbactam was equally as effective as cefoperazone/sulbactam with respect to the clinical cure rate (36.43 vs. 39.55%), the efficacy rate (85.07 vs. 79.84%) and the bacterial eradication rate (83.58 vs. 83.72%). In RTIs, the clinical cure rate (27.69% in the ceftriaxone/sulbactam group vs. 20% in the cefoperazone/sulbactam group) and the efficacy rate (76.92% in the ceftriaxone/sulbactam group vs. 70.77% in the cefoperazone/sulbactam group) were comparable. Similar results were observed in the treatment of UTIs. There were no statistically significant differences in any of the rates for the two drugs. Both drugs were more efficacious when treating UTIs, possibly due to the higher bacterial eradication rates. The cure rates were relatively low for both drugs, especially when treating RTIs, and were far lower than previously reported rates [10,11]. Recently, the clinical response in a multicentre clinical trial of ceftriaxone/sulbactam in lower respiratory tract infections in India indicated an average cure rate of 77.1% (unpublished data). The lower cure rates in our study are likely due to the patient conditions and the occasional inconsistence of *in vitro* activity (susceptible) and clinical outcomes (suboptimal) for these isolated resistant strains.

The *in vitro* susceptibility study of the isolates demonstrated the remarkable antibacterial activity of ceftriaxone/sulbactam, with an overall susceptibility rate of 84.88%. The MICs of all of the microbial strains tested were significantly reduced with ceftriaxone/sulbactam compared with ceftriaxone alone, which is consistent with results reported by other investigators [3,4,6,7]. All of the bacteria isolated in this study were β-lactamase-positive, although we unfortunately did not determine the specific types of β-lactamases from these isolates. Members of the family Enterobacteriaceae are among the most important human bacterial pathogens. These microbes comprise approximately 65% of all Gram-negative bacteria and 47% of all of the isolates identified in hospital laboratories in China [13]. The production of β-lactamases and the emergence and proliferation of extended-spectrum β-lactamases (ESBLs) are increasing the antimicrobial resistance of many species of Enterobacteriaceae and causing the dissemination of multidrug-resistant (MDR) strains [14,15]. CTX-M enzymes are the dominant ESBL type in China [16]. A previous study has demonstrated the *in vitro* efficacy of ceftriaxone/sulbactam against bacteria harbouring these types of enzymes [7,17].

Notably, ceftriaxone alone does not possess adequate activity against *A. baumannii* and *P. aeruginosa*. The combination restores a degree of antimicrobial activity against these species. Therefore, ceftriaxone/sulbactam offers a potential alternative to cefoperazone/sulbactam for the treatment of infection caused by these intrinsically resistant species.

Both of the combinations have favourable safety records and are well-tolerated. The patients receiving ceftriaxone/sulbactam exhibited an insignificant increase in drug-related clinical adverse events compared with patients receiving cefoperazone/sulbactam (10.42 vs. 7.09%, *P* = 0.3212). Among the adverse events, gastrointestinal side effects, nervous system symptoms, hepatic function and routine blood test abnormalities were the most frequently encountered. Although one severe adverse event of nephrorrhagia occurred in a patient with a recent pyelolithotomy who had received the ceftriaxone-sulbactam treatment, an investigation verified that the effect was most likely unrelated to the drug itself. Nearly all of the side effects were mild and well tolerated, which suggested that ceftriaxone/sulbactam could be safely employed in clinical settings for the treatment of the infections studied.

Due to the limitations of clinical trials, most of the cases investigated were from community-acquired infections in which the general resistance rate of bacteria is low. This low rate contributed to the 5 years that were required to complete this trial. Based on the characteristics of the β-lactam/β-lactamase-inhibitor combination, ceftriaxone/sulbactam should be used for the treatment of multidrug-resistant bacterial infections. More data are required for the treatment of nosocomial infections.

## Conclusion

Ceftriaxone/sulbactam is as effective and well-tolerated as cefoperazone/sulbactam for the treatment of intermediate and severe bacterial infections caused by resistant strains.

## Competing interests

The authors declare that they have no competing interests.

## Authors’ contributions

XJX was involved in the protocol designing, collection and analysis data for this study. LJ was involving in drafting this manuscript. XYX was involved in the collection and analysis of data for this study, as well as revising the content. BJ was involved in the protocol design, collection of data for this study, the analysis and interpretation of these data, and in drafting and revising the content of this manuscript. WXH was involved in the protocol design and has given final approval of the version to be published. CZL was involved in microbiological assays. CZW, LYZ, XZS, XHT, YJH, YKZ and WLZ were involved in the collection of data for this study. All authors read and approved the final manuscript.

## References

[B1] Xiao YH Wang J Yan Z Qi HM Xy L Zhao CY Mohnarin of 2008 Surveillance results of national bacterial drug resistanc Chin J Clin Pharmacol Therap 2010 12 16 2377 2383

[B2] Akova M Sulbactam-containing beta-lactamase inhibitor combinations Clin Microbiol Infect 2008 12 Suppl 1 185 188 18154545 10.1111/j.1469-0691.2007.01847.x

[B3] Shrivastava SM Kumar S Chaudhary M Ceftriaxone-sulbactam combination: microbial analysis by variation of ratios and comparative disc diffusion Curr Res Bacteriol 2009 12 50 55 10.3923/crb.2009.50.55

[B4] Payasi A Chaudhary M Gupta A Dwivedi VK Bhatnagar A Pharmacokinetic study of sulbactomax J Toxicol Sci 2010 12 459 464 10.2131/jts.35.459 20686332

[B5] Yonghong X Yuan L Zisheng K Jan L Tolerability and pharmacokinetics of multiple dose ceftriaxone/sulbactam injection in chinese Chin J Clin Pharmacol Therap 2006 12 458 506

[B6] Shrivastava SM Saurabh S Rai D Dwivedi VK Chaudhary M *In Vitro* microbial efficacy of sulbactomax: a novel fixed dose combination of ceftriaxone sulbactam and ceftriaxone alone Curr Drug Ther 2009 12 73 77 10.2174/157488509787081840

[B7] Shahid M Singhai M Malik A Shukla I Khan HM Shujatullah F *In vitro* efficacy of ceftriaxone/sulbactam against Escherichia coli isolates producing CTX-M-15 extended-spectrum beta-lactamase J Antimicrob Chemother 2007 12 187 188 Epub 2007 May 8 10.1093/jac/dkm131 17491002

[B8] Haozhu C Practice of Internal Medicine 1998 10 Beijing: The People’s Medical Publishing House

[B9] Clinical and Laboratory Standards Institute Performance standards for antimicrobial susceptibility testing; Twentieth informational supplement. CLSI documents M100-S15~19 2005-2009 London: CLSI

[B10] Li JT Lu Y Hou J Chen YF Miao JZ Jia YX Sulbactam/cefoperazone versus cefotaxime for the treatment of moderate-to-severe bacterial infections: results of a randomized, controlled clinical trial Clin Infect Dis 1997 12 498 505 10.1093/clinids/24.3.498 9114206

[B11] Koga H Tomono K Hirakata Y Kohno S Abe K Kawamoto S Clinical evaluation of sulbactam/cefoperazone for lower respiratory tract infections. Correlation between the efficacy of sulbactam/cefoperazone and beta-lactamase Jpn J Antibiot 1996 12 800 807 9053534

[B12] Drawz SM Bonomo RA Three decades of beta-lactamase inhibitors Clin Microbiol Rev 2010 12 160 201 10.1128/CMR.00037-09 20065329 PMC2806661

[B13] Zhu DM Wang F Hu FP Jiang XF Ni YX Sun JY CHINET 2010 surveillance of bacterial resistance in China Chin J Infect Chemother 2011 12 321 329

[B14] Gray KJ Wilson LK Phiri A Corkill JE French N Hart CA Identification and characterization of ceftriaxone resistance and extended-spectrum beta-lactamases in Malawian bacteraemic Enterobacteriaceae J Antimicrob Chemother 2006 12 661 665 10.1093/jac/dkl037 16537341

[B15] Bhattacharjee A Sen MR Prakash P Anupurba S Role of beta-lactamase inhibitors in enterobacterial isolates producing extended-spectrum beta-lactamases J Antimicrob Chemother 2008 12 309 314 Epub 2008 Jan 3 18174199 10.1093/jac/dkm494

[B16] Liu W Chen L Li H Duan H Zhang Y Liang X Novel CTX-M {beta}-lactamase genotype distribution and spread into multiple species of Enterobacteriaceae in Changsha, Southern China J Antimicrob Chemothe 2009 12 895 900 Epub 2009 Mar 18 10.1093/jac/dkp068 19297379

[B17] Fantin B Pangon B Potel G Caron F Vallée E Vallois JM Activity of sulbactam in combination with ceftriaxone *in vitro* and in experimental endocarditis caused by Escherichia coli producing SHV-2-like β-lactamase Antimicrob Agents Chemother 1990 12 581 586 10.1128/AAC.34.4.581 2188586 PMC171647

